# A path forward in the debate over health impacts of endocrine disrupting chemicals

**DOI:** 10.1186/1476-069X-13-118

**Published:** 2014-12-22

**Authors:** R Thomas Zoeller, Åke Bergman, Georg Becher, Poul Bjerregaard, Riana Bornman, Ingvar Brandt, Taisen Iguchi, Susan Jobling, Karen A Kidd, Andreas Kortenkamp, Niels E Skakkebaek, Jorma Toppari, Laura N Vandenberg

**Affiliations:** University of Massachusetts Amherst, Amherst, MA USA; Swedish Toxicology Sciences Research Center (Swetox), Forskargatan 20, SE-151 36 Sodertalje, Sweden; Norwegian Institute of Public Health, Oslo, Norway; University of Southern Denmark, Odense, Denmark; School of Health Systems and Public Health, University of Pretoria, Pretoria, South Africa; Uppsala University, Uppsala, Sweden; National Institute for Basic Biology, Okazaki, Japan; Brunel University, London, UK; University of New Brunswick, Saint John, New Brunswick, Canada; Brunel University, London, UK; Rigshospitalet, University of Copenhagen, Copenhagen, Denmark; University of Turku, Turku, Finland; University of Massachusetts Amherst, Amherst, MA USA

**Keywords:** Endocrine disruptor, UNEP, WHO, State of the science

## Abstract

Several recent publications reflect debate on the issue of “endocrine disrupting chemicals” (EDCs), indicating that two seemingly mutually exclusive perspectives are being articulated separately and independently. Considering this, a group of scientists with expertise in basic science, medicine and risk assessment reviewed the various aspects of the debate to identify the most significant areas of dispute and to propose a path forward. We identified four areas of debate. The first is about the definitions for terms such as “endocrine disrupting chemical”, “adverse effects”, and “endocrine system”. The second is focused on elements of hormone action including “potency”, “endpoints”, “timing”, “dose” and “thresholds”. The third addresses the information needed to establish sufficient evidence of harm. Finally, the fourth focuses on the need to develop and the characteristics of transparent, systematic methods to review the EDC literature. Herein we identify areas of general consensus and propose resolutions for these four areas that would allow the field to move beyond the current and, in our opinion, ineffective debate.

## Background

Several recent publications have reflected intense debate concerning the potential health effects of “endocrine disrupting chemicals” (EDCs). For example, Kortenkamp et al. [1] produced a “State of the Art” of EDCs document under contract from the European Commission, about which there was a critical editorial [2], and a response [3]. Likewise, Vandenberg et al. [4] conducted a major review of the evidence for low-dose effects of chemicals on the endocrine system, about which there was a critical editorial [5], and a rebuttal [6]. More recently, a group of toxicology journal editors [7] wrote an open letter to the then science advisor to the European Commission concluding that the Commission was proposing an approach that lacks “adequate scientific evidence” [8]; this letter was criticized by a number of scientists in two separate responses [9, 10]. In 2010, the United Nations Environmental Programme (UNEP) and the World Health Organization (WHO) assembled a working group of 16 scientists from 10 countries to write a review on the state of the science of endocrine disruptors, with specific content added by 9 other experts [11]. Twenty-three independent scientists from 12 countries reviewed the semi-final draft, and the final version was reviewed and approved by UNEP and WHO scientists prior to its publication in early 2013. Like before, a group of authors published a critical editorial of this document [12] and many of the same criticisms have been found elsewhere [13].

Thus, in large measure, the current “debate” has taken the form of two apparently mutually exclusive perspectives, but perhaps revolving around issues that may in fact not be disputed between the groups. To illustrate this, the then Chief Scientific Advisor to the President of the European Commission (Professor Anne Glover) held a meeting between representatives of the two opposing perspectives [7–9], and there was surprising consensus on issues that Dietrich et al. had originally contested [13]. Because the critical analysis of the UNEP/WHO report [11] by Lamb et al. [12] is the longest and most detailed, and because it covers the same issues expressed in other critical reviews, we use this as the focus of our current analysis. Our goal is to review aspects of the debate as revealed in these publications, identify areas of disagreement, and propose a common path forward.

### The role of definitions: is everyone talking about the same thing?

**Endocrine Disrupting Chemical (EDC)**

Several groups have proposed definitions for an EDC. These definitions have been reviewed previously [14, 15] and are included in Table 1. For example, the definition proposed by the WHO/IPCS document of 2002 [16] is: “An endocrine disrupter is an exogenous substance or mixture that alters function(s) of the endocrine system and consequently causes adverse health effects in an intact organism, or its progeny, or (sub) populations”. This definition employs certain terms (“function(s) of the endocrine system”, “endocrine system”, “adverse effect”) that have been inconsistently applied and, therefore, have created the appearance of a dispute where none may exist.

**Table 1:** **Definitions of endocrine disruptors**

Date	Agency	Definition
1996	US EPA^1^	An exogenous agent that interferes with the production, release, transport, metabolism, binding, action, or elimination of natural hormones in the body responsible for the maintenance of homeostasis and the regulation of developmental processes.
1996	EU^2^	An exogenous substance that causes adverse health effects in an intact organism, or its progeny, secondary to changes in endocrine function. A potential ED is a substance that possesses properties that might be expected to lead to endocrine disruption in an intact organism.
1998	The Environment Agency	An endocrine disruptor is an exogenous substance that causes adverse health effects in an organism, or its progeny, consequent to endocrine function.
1999	National Academy of Science	The term hormonally active agents (HAAs) is used to describe substances that possess hormone-like activity regardless of mechanism. Convincing evidence that an HAA can affect the endocrine system would be its ability to bind to classic hormone receptors and promote measureable responses such as the induction of hormone-responsive genes or gene products. However, chemicals can disrupt hormonal processes by a variety of other mechanisms.
2000	The Royal Society	EDCs are substances which may interfere with normal function of the endocrine (hormone) system of human and animals, since many of them mimic the structure of natural hormones produced in the body.
2000	German Consultative Study	Substances able to disrupt endocrine processes with the potential for impairing development and reproduction or increasing the risk of cancer.
2002	WHO/IPCS^3^	An exogenous substance or mixture that alters function(s) of the endocrine system and consequently causes adverse effects in an intact organism, or its progeny, or (sub)populations. A potential endocrine disruptor is an exogenous substance or mixture that possesses properties that might be expected to lead to endocrine disruption in an intact organism, or its progeny, or (sub)populations.
2012	Endocrine Society^4^	An exogenous chemical, or mixture of chemicals, that interferes with any aspect of hormone action.

^1^United States Environmental Protection Agency [17].

^2^European Union at the Weybridge Conference [18].

^3^World Health Organization/International Programme on Chemical Safety [16].

^4^ [15].

The first area of debate is the term “function(s) of the endocrine system”. Some authors use this term to mean a change in hormone concentrations in the blood. Therefore, an EDC would include a candy bar which, when eaten, would cause insulin secretion, thereby altering the “function” of an endocrine system. This issue was highlighted by Nohynek et al. [14], who wrote, “… could a single Chinese meal or a cup of coffee wreak havoc with our endocrine systems? Does this assumption appear reasonable?” Obviously, no one would propose that a candy bar or a meal of Chinese food would constitute disruption of the function of the endocrine system; but they do change hormone concentrations in the blood. This concept can be found in many publications because of the way “endocrine function” is being interpreted.

In contrast, recent research demonstrates that EDCs can change the responses of the endocrine system to normal events [19]. For example, studies have shown that the female hormone, 17β-estradiol, can increase insulin production in the pancreas, but that the chemical bisphenol A can overstimulate the estrogen receptor potentially leading to insulin resistance – an important component of type 2 diabetes [20]. In addition, recent evidence also shows that EDCs can interfere with the effects of hormones in tissues in a manner that is not reflected by changes in hormone concentrations in the blood [21]. In recognition of this, the Endocrine Society (the largest professional society of clinical and research endocrinologists) offered a biologically-based definition of an endocrine disruptor: “An ED is an exogenous chemical, or mixture of chemicals, that interferes with any aspect of hormone action” [15]. In this view, an EDC would be a chemical that changes the way the pancreas responds to the candy bar (or a meal of Chinese food), or that blocks the ability of insulin concentrations to rise or to act to lower glucose. By focusing on “hormone action” instead of “endocrine function”, a candy bar (or a meal of Chinese food, or a coffee) would not fit this definition of an EDC because it does not interfere with hormone action.

We should note here that by hormone “action” we mean “hormone receptor activation” that leads to developmental or physiological effects. Hormone receptors are proteins that mediate the effect of the hormone on a cell; EDCs can interfere with hormone action either by interacting directly with a receptor, or by interfering with the normal delivery of the hormone to the receptor [19]. By “normal delivery”, we mean that a chemical can interfere with hormone synthesis, release, transport in blood or across membranes, metabolism or clearance. In short, any process that affects the ability of the hormone to come into contact with the receptor to impact “hormone action”. In addition, some chemicals have been shown to interact with a hormone receptor and cause it to exert a different action [22]. This kind of mechanism will be particularly insidious because it will produce effects that do not faithfully recapitulate an agonist or antagonist action.

The various definitions of an EDC proposed by regulatory agencies are not likely to change. And, in principle, the term “endocrine function” is reasonable as long as it is viewed in terms of hormone action and not simply of hormone concentrations in blood. Thus, the current debate would be greatly advanced if we could agree that what is meant by “endocrine function” is, in fact, “hormone action” (in the sense defined above).

### Endocrine system

A second, related concept – and one that is a major contributor to the debate – is the way the endocrine system is understood, and the way its role in human health and disease is envisioned. For example, Lamb et al. [12] state that, “… the endocrine system is specifically designed to respond to environmental fluctuations and such homeostatic responses generally are considered normal, adaptive, and necessary as long as they are transient and within the normal homeostatic range”. Likewise, Dietrich et al. [8] state that, “… endocrine systems play a fundamental role in the physiological response to changes in the environment with the aim of keeping an organism’s biology within the homeostatic space. It is the task of the toxicologists to make the distinction between those effects that are within this adaptive range and effects that go beyond the boundaries of this space and thus can be called adverse”.

There are two elements of this definition and perspective of the endocrine system that contribute to the debate. First is the concept that the endocrine system is specifically designed to respond to environmental fluctuations. While the endocrine system does respond to physical stressors in the environment to maintain (e.g.) body temperature, water and ion balance, cardiovascular function etc., the endocrine system also plays essential roles in growth and development, intermediary metabolism and reproduction [23]. Thus, the perspective that the endocrine system’s primary role is to maintain the organism within homeostatic space conflicts with primary texts of endocrinology, and does not appear to take into consideration the essential role of hormones in brain development [24], in sexual differentiation (e.g., [25]), in establishing the set-point for metabolism or stress responses later in life [26, 27] and others. This conflict between “homeostasis” and “developmental effects” accounts for a significant amount of the debate. In addition, the second element is that the Lamb et al. and Dietrich et al. perspective of the endocrine system appears to imply that environmental chemicals represent a natural, physical stressor such as temperature, water and food availability, etc., to which the endocrine system can respond in an adaptive way. In contrast, research in the field of EDCs establishes clearly that industrial chemicals interfere with hormone action in ways that cannot be considered similar to natural environmental stressors and are often irreversible [15, 19].

### Adverse effects

A third related issue is the term “adverse effect”. As described by Nohynek et al. [14], “All current definitions agree that the definition of an ‘adverse health effect’ means toxicity, i.e. pathology or functional impairment. Therefore, only a substance that produces toxicity in an intact organism via a hormonal or hormone-like mechanism represents a genuine ED.” This definition and a similar one used by Lamb et al. [12] deviate somewhat from the IPCS wording [28]: “change in morphology, physiology, growth, development, reproduction or life span of an organism, system, or (sub) population that results in an impairment of functional capacity, an impairment of the capacity to compensate for additional stress, or an increase in susceptibility to other influences.” If we accept all of these definitions of an adverse effect, then it becomes even more important to focus on “hormone action” rather than “endocrine function”. There is likely to be widespread agreement that an EDC would produce an adverse health effect (i.e., toxicity) if and only if it interferes with hormone action, which may or may not be related to a change in hormone concentrations in the bloodstream.

Because the process to determine whether an endpoint is “adverse” is not transparent, there remains great concern about whether this term is applied consistently [29], as well as whether it acknowledges scientific measurements that map to, or predict, human diseases. In addition, it will be critical to reach agreement about whether “adverse effects” only apply to individual clinical symptoms (e.g., death or cancer) or whether an increase in disease burden in a population is included. It is important to note that because there are several accepted definitions of “adverse effect”, differences between these definitions will likely influence which scientific studies are included in a particular risk assessment (e.g., Table 2).

**Table 2:** **Definitions for ‘adverse effect’ and their origins**

Origin	Definition	Reference
US EPA	“a biochemical change, functional impairment, or pathologic lesion that affects the performance of the whole organism, or reduces an organism’s ability to respond to an additional environmental challenge”	[30]
IPCS/OECD	“a change in morphology, physiology, growth, development or lifespan of an organism which results in impairment of functional capacity or impairment of capacity to compensate for additional stress or increase in susceptibility to the harmful effects of other environmental influences”	[28]
US FDA	none	
EFSA	none	
Nohynek et al.	“toxicity, i.e. pathology or functional impairment”	[14]

Thus, to move forward, it is essential to define our language related to EDCs. First, it is important that we realize that adverse outcomes of chemical exposure – however they are defined – can be mediated by an endocrine mechanism if and only if the chemical interferes with hormone action. This may be reflected by changes in hormone concentrations in the blood, but we should not interpret “endocrine function” as a “change in hormone concentration”. Second, our definition of the endocrine system must take into consideration the developmental and organizational effects of hormones. It makes little sense scientifically to have groups of authors who have never studied the endocrine system create new definitions that are not recognized by scientists who have developed the knowledge base for the field. Finally, we must agree on what constitutes an “adverse effect”. Several regulatory agencies have defined an “adverse effect” and these can reasonably be the basis for this discussion.

### Features of hormone action: which elements of hormone action are most relevant to the EDC debate?

Several features of hormones and hormone action are the focus of this debate, but different authors emphasize different features. Which are the most important? This part of the EDC debate is more nuanced because hormone action is complex, but it generally falls under the categories of “potency”, “endpoints”, “timing” and “thresholds”.

### Potency

Pharmacologists define potency as a measure of a substance’s activity, expressed as the amount of a substance that is required to produce a specific effect at a specific level of intensity. In the field of toxicology, this could mean the dose that induces death in 50% of treated animals (the LD50) or the dose that reduces body weight by 20%. It is important to recognize that a chemical will have a different activity (i.e., potency) on different specific effects (i.e., endpoints). For example, lead is much more potent at affecting the developing brain than it is at causing death. This means that a discussion of a chemical’s potency must include mention of the specific effects being considered.

In the study of EDCs, potency is often used to compare the doses required to induce a specific response (e.g., a significant change in uterine weight) for a test substance compared to a dose of a hormone (for example, the natural estrogen, 17β-estradiol). Nohynek et al. [14] compared the potency of a variety of chemicals to the synthetic estrogen 17α-ethinylestradiol (EE) and conclude that comparing EE with benzylparaben (BP) is like comparing the power of an aircraft carrier (EE) with that of a child on a bicycle (BP). This kind of general comparison is visually impressive but, without a discussion of the endpoints being employed for the comparison and whether that endpoint is sensitive or insensitive to the hormone, it does not advance our understanding of potency. Recent evidence demonstrates that there are EDCs that have been described as “weak estrogens” in some contexts that are equipotent to 17β-estradiol in other contexts [15]. Thus, to move this discussion forward, we must agree on the endpoints that are important to consider as metrics of “potency” and recognize that as new science becomes available, our perception of the relative potency of a chemical may change.

### Thresholds

The threshold model in toxicology predicts that there will be no effect of a chemical below a ‘threshold’ of exposure, but there will be effects at doses above. This concept is the basis upon which decisions of chemical safety are determined, when toxicity testing has not been performed at doses that mimic human exposures [31, 32]. Although simple to imagine, this concept is actually highly complex for several reasons. First, the existence of dose thresholds cannot be proven or disproven based on experimental observation because measured effects themselves have a limit of detection that will obscure the observation of a threshold, if it exists [33]. Second, identifying a threshold in the human population is confounded because not all people are equally sensitive to the effects of a chemical; there would be a graded response to a chemical thereby obscuring the observation of a threshold, if there is one. Slob, as well as the authorship of the National Academy of Sciences document “Science and Decisions”, have argued that it is impossible to define thresholds at the population level for any endpoint (including cancer and non-cancer effects) [34]. Finally, because different endpoints are differentially sensitive to hormones, it is unrealistic to imagine a single threshold value, if they exist, for all endpoints of an EDC.

The belief in a dose threshold is therefore derived from the way one imagines that an EDC acts to produce an adverse effect, rather than being evidence-based. We are a long way from a full understanding of the endocrine system and of the ways hormones act; thus, it stands to reason that we are also a long way from a full understanding of the ways EDCs act. To move this debate forward, we must acknowledge first that dose thresholds are impossible to prove or disprove experimentally, as indeed has been recognized during a meeting of the participants in the public debate, with the then Chief Scientific Advisor to the EU Commission President, Professor Anne Glover [14]. Second, it is essential to appreciate that the discussion must be based on the recognition of the limits of our understanding of endocrine systems and hormone actions. This will require more humility than hubris.

### Endpoints

The term “endpoint” is broad and typically refers to a measure of disease, a symptom, or a predictor of disease that is being evaluated in response to chemical exposure. Because hormones have roles in the development and regulation of virtually every system and organ in the body, the range of “endpoints” that may be affected by an exogenous hormone or EDC is extensive.

A large part of the EDC debate is on the various endpoints that have been used in studies to assess chemical effects. One type of study, guideline studies, uses prescribed methods that have been agreed upon by committees and validated to demonstrate their reproducibility [35]. Although there are positive aspects to guideline studies (i.e., reproducible methods), even validated laboratories have difficulties replicating the effects of specific compounds at specific doses [36]. Furthermore, guideline endpoints – primarily body and organ weight – have been shown to be significantly less sensitive than the endpoints examined by specialists who study effects of EDCs on a particular developmental or physiological process.

Moreover, guideline endpoints do not map explicitly to a specific human disease or dysfunction [15]. They also do not cover the entirety of the diseases that can be affected by EDCs; for example, there is no guideline assay to assess whether a substance alters the response of an organism to a hormonal or carcinogenic challenge, a high fat diet, stress, or other environmental factors. Yet, these environmental factors are known to contribute to many human diseases including cancers, reproductive disorders, metabolic disorders, and others. Moreover, there are no guideline endpoints that predict the effects of chemical exposures on asthma, diabetes, or many of the chronic diseases that plague human populations today.

Although there is extensive evidence that non-guideline studies, examining non-guideline endpoints, have identified adverse effects of EDCs [4, 15], these are often not accepted in chemical safety assessments for reasons that have little to do with their predictive power and more to do with compliance to specific record-keeping methods [37]. To develop more predictive and comprehensive endpoints is a complex issue and beyond the scope of this review. However, a collaboration currently underway between the National Institute of Environmental Health Science (NIEHS), the National Toxicology Program (NTP), and the Food and Drug Administration (FDA) is comparing the sensitivity of guideline and non-guideline endpoints in the same animals exposed to EDC treatment [38]. This so-called “CLARITY-BPA” study also represents a paradigm that could easily incorporate a strategy to validate new and more sensitive endpoints into guideline studies [39].

### Timing

From the perspective of endocrinology, the timing of exposure is one of the most important influences on the effects of a hormone or an EDC [40]. This issue not only derives its importance from the recognition of hormone effects in development, but also from the importance of discussions of “adverse effects” and “potency”. More specifically, hormones produce effects during development that can either have direct effects on the adult offspring or life-long effects on the way the individual responds to various hormones as adults. For example, thyroid hormone action during fetal development is necessary for normal brain development; thyroid disruption or thyroid hormone insufficiency during development can reduce cognitive function (e.g., global intelligence) throughout life [24]. However, thyroid disruption or thyroid hormone deficiency in adults will have different effects, many of which are reversible [41]. Likewise, androgens are responsible for the male external (and internal) reproductive structures; thus, a genetic male with a mutation that completely prevents androgen action will be phenotypically female [42]. In contrast, a deficiency in androgen action in adult males will have completely different effects.

Also, the impacts of endocrine disrupting exposures during development may not be observed until much later in life. In the case of diethylstilbestrol (DES), cancers of the reproductive tract did not appear in the female offspring of women prescribed DES until after puberty [43]. Likewise, because testicular cancer is of fetal origin but does not appear until after puberty, there is concern that endocrine dysfunction or disruption during fetal development can also lead to a delayed adverse effect [44]. Indeed it is becoming clear from animal studies that many complex non-communicable diseases typically experienced in adulthood (cancers, metabolic syndrome, infertility, etc.) have their origins during development that can be produced by a variety of environmental stressors including EDCs [45].

### “Low dose” effects

Hormones produce effects at extremely low concentrations under normal conditions [46]. Natural hormones typically circulate in the body at part-per-billion and part-per-trillion concentrations; only a small fraction of the total concentration of circulating steroid hormone in blood is in a form that is free to impact tissues [4]. There is a significant literature about the impact of EDCs at a “low dose” [47]. In the study of EDCs, the term “low dose” is used in different ways and typically to distinguish studies that examine effects: (1) below the doses used in traditional toxicology studies, i.e., doses below the no or low adverse effect level (NOAEL or LOAEL); (2) at doses in the range of typical human exposures; or (3) at doses in animals that replicate the circulating concentrations of a substance in humans [4].

There is desire among some practitioners in the field to simplify this language and use only a single definition for “low dose”, but a consensus has not yet been reached [48]. In 2002, an expert panel assembled by the NTP and the US EPA summarized the evidence for low dose effects of four EDCs, which were found to have reproducible and consistent effects on specific endpoints [49]. This panel included scientists from academia, government laboratories, and industry; thus, suggestions that there is a lack of consensus on the presence of “low dose effects” [5, 12], or that low dose effects are “hypothetical… highly improbable, if not impossible” [14] are inaccurate and outdated at best. A series of reviews, published in 2012 and 2013, updated the evidence for the effects of EDCs at a “low dose”, and revealed low dose effects for more than two dozen EDCs beyond those considered by the 2002 NTP/EPA panel [4, 6]. These issues were also discussed at a 2012 international workshop attended by governmental, industry and academic scientists [48]. To resolve this issue, we will first have to agree to use consistent language; all three definitions of “low dose” are valid, but we must ensure that any debate is focused on the same definition. Second, we will have to agree on endpoints that are considered “adverse” because one argument is that while there are effects of EDCs at “low doses” by any definition, these effects are not adverse.

### What constitutes “sufficient evidence” of harm for regulatory agencies to take action?

In his presidential address, Sir Austin Bradford Hill made the following observation that resonates true today:

“Finally, in passing from association to causation I believe in ‘real life’ we shall have to consider what flows from that decision. On scientific grounds we should do no such thing. The evidence is there to be judged on its merits and the judgment (in that sense) should be utterly independent of what hangs upon it - or who hangs because of it.”

Studies in environmental epidemiology aim to determine whether environmental factors (like EDC exposures) are associated with a disease or dysfunction within a population. Unlike controlled, randomized clinical trials, exposures to EDCs are almost always uncontrolled and other factors (such as the long latency between exposure and disease outcome) can complicate this type of study. Moreover, chemical exposures do not occur in isolation and, even in newborns, there are literally dozens of chemicals found in the bloodstream [50]. Considering these factors, it has been strongly debated whether environmental epidemiology studies can show causal relationships between exposures and disease as Bradford Hill envisaged the elements of data contributing to a conclusion of a causal association [51].

Therefore, a significant part of this debate centers on the definition of “causation” and the methods employed to determine causal relationships. Lamb et al. [12] define “causation” as follows: “To say that an agent causes an adverse effect means that the agent interacts with an organism to produce changes that lead to adverse effects that would not have occurred had the agent not been present.” This definition may, for example, exclude cigarette smoking as a cause of lung cancer because not all lung cancers are attributable to smoking. Likewise, in an experiment designed to identify the dose at which 50% of the animals die (i.e., LD50), both living and dead animals received the same dose of agent; Lamb et al.’s definition may not allow one to conclude that the agent caused 50% of the animals to die because the other 50% was exposed to the chemical but did not die.

The nature of causation is a core issue for science, and there is a great deal written on this subject [52]. It is possible that Lamb et al. intended to say that a toxic chemical *causes* an adverse effect when it increases the frequency or intensity – over that of controls – of that “adverse endpoint”. Yet, even if this were Lamb et al.’s intended definition for causation, it would preclude drawing conclusions about causal relationships from any environmental epidemiology studies, which by nature are not controlled. In the field of environmental epidemiology, it is generally recognized that, in principle, an agent causes an adverse effect when some proportion of the disease burden is attributable to exposure to that agent. The elements for establishing causation proposed by Bradford-Hill almost 50 years ago [51] provide a framework by which a causal relationships can be deduced. Yet, these elements depend on a level of expert judgment and appear to be employed by different groups in different ways. Therefore, it is important to clearly evaluate how the various elements as articulated by Hill fit the EDC debate (Table 3).

**Table 3:** **Bradford Hill Elements of Data Contributing to a Causal Association and EDCs**

Hill Elements ^1^	Application to EDCs
*Strength of Association*	
The examples used were testis cancer in chimney sweeps and lung cancer in smokers. In both examples, the strength of the associations were made by comparing death rates in a control group (men who were not chimney sweeps and non-smokers, respectively).	There are no groups of people unexposed to EDCs. Moreover, no one is exposed to a single chemical. Finally, endocrine diseases and disorders are clearly multicausal. Thus, the concept of strength of the association must be adjusted as it is applied to EDCs.
*Consistency*	
The concept is that multiple studies should observe the same relationships between exposure and outcome.	In principle, there should also be consistent observations between relationships of interest. However, there are at times modifying factors that can change this. For example, perchlorate exposure is inversely related to serum thyroid hormone in populations with low iodine intake or in those who smoke cigarettes. However, this is not the case in populations with high iodine intake and/or who do not smoke.
*Specificity*	
The example was that of nickel refiners of South Wales with a high incidence of cancer of the lung or nose. The specificity of this relationship could be used as evidence of causation. However, Hill cautioned about making too much of the specificity of the relationship and concluded that, “In short, if specificity exists we may be able to draw conclusions without hesitation; if it is not apparent, we are not thereby necessarily left sitting irresolutely on the fence.”	The specificity of relationships of interest with EDCs must be evaluated carefully because hormone systems are involved in a great many processes and this is life-stage specific. For example, androgens play an important role in development of the male reproductive system in the fetus, but in the adult, androgens are related to different processes in men and women. Likewise, transient hypothyroidism during fetal development can lead to lower IQ and attention deficit, but transient hypothyroidism in the adult can lead to weight gain that is reversible.
*Temporality*	
Hill’s concept was to be cautious about the temporal relationship of associations with particular attention to the question of which element of the dyad came first? For example, do particular dietary habits lead to disease, or does the disease predispose those affected to prefer a specific diet?	The temporal relationship between exposure to an EDC and a specific endocrine-mediated adverse outcome may be quite complex. The classic example is that of DES exposure during fetal life and the production of reproductive tract cancer 20 years later (long after DES was gone). This relationship was observed because women were prescribed DES and there were specific records of exposure. This will not likely be the case for non-accidental exposures to EDCs. Thus, “temporality” may be important, but it may not be a concurrent relationship.
*Biological Gradient*	
Hill noted that the linear increase in the death rate from lung cancer with number of cigarettes smoked daily added greatly to the simple evidence that the cancer rate was higher in smokers than non-smokers. But he didn’t discount a relationship in which the death rate is higher in people who smoke fewer cigarettes per day.	The shape of the dose–response is important for EDCs, but there may be more variability depending on the mechanism of disruption. For example, perchlorate should produce a typical S-shaped dose–response curve on thyroid hormone concentrations in the human population because it is a competitive inhibitor of iodine uptake into the thyroid gland. In contrast, BPA is likely to produce more of a “square wave” dose–response curve because it is an indirect antagonist on the thyroid hormone receptor.
*Plausibility*	
Hill insisted that “it will be helpful” if the causation we suspect is biologically plausible. However, we cannot demand this. In short, the association we observe may be one new to science or medicine and we must not dismiss it too light-heartedly as just too odd.	Likewise for EDCs, biological plausibility will likely strengthen our confidence in the causal nature of relationships of interest. Moreover, our knowledge of hormone actions will likely drive us to evaluate specific relationships. However, there is a great deal we have to learn about the endocrine system, and requiring complete knowledge of the endocrine mechanism mediating a relationship of interest is unrealistic.
*Coherence*	
Hill reasoned that the interpretation of a causal relationship between exposure and outcome should not conflict with generally known facts of the natural history and biology of the disease.	Coherence is also important for EDCs. Thus, the interpretation of causation should not conflict with generally known facts of the biology of the endocrine system under study.
*Experiment*	
Hill reasoned that occasionally, confidence in a conclusion of causality could be strengthened by changing elements of the environments and observing a predicted change. For example, dust in the workplace could be reduced, oil changed, work conditions altered. He did not include animal or biochemical experiments.	For EDCs, animal and biochemical experimental evidence must be integrated with (or without) epidemiological data to consider that a chemical may produce an adverse outcome through an endocrine mechanism. This is a novel component of assessing the evidence and the logic guiding this has not been formally validated. Because of the complexity of hormone action, such experiments need to be properly designed with positive and negative controls, and must be properly interpreted based on principles of endocrinology.
*Analogy*	
Hill reasoned that known causal relationships can reasonably be extended to other relationships that have similar characteristics. His example was that with the effects of thalidomide and rubella being known, it would be more likely to be reasonable to accept slighter but similar evidence with another drug or viral disease in pregnancy.	Likewise, it is reasonable in the EDC field to extend this to include analogous endpoints. For example, if we observe a relationship between phthalate exposure and anogenital distance in newborn boys, we can reasonably extend this relationship to other androgen-dependent endpoints. Moreover, if we know that a chemical has antiandrogenic properties *in vitro*, it is reasonable to tailor the endpoints that are evaluated *in vivo* to androgen-sensitive endpoints. Likewise, if we observe a relationship between PCB exposure and the expression of thyroid hormone-responsive genes in the placenta, we can reasonably extend this to thyroid hormone action in tissues we cannot obtain, such as the fetal/neonatal brain. And if we know that PCBs have anti-thyroid properties, we should evaluate thyroid-sensitive endpoints.

^1^These elements are taken from: Hill AB. The Environment and Disease: Association or Causation? Proc R Soc Med. 1965 May;58:295–300 [51].

These considerations seem to have been ignored when Lamb et al. [12] criticized the UNEP/WHO report [10] for not adopting the Bradford-Hill approach. In fact, the UNEP/WHO report presents a detailed discussion of the challenges associated with the Bradford-Hill approach as a tool for judging causality within the context of EDCs. These problems were recognized by Bradford-Hill himself [43] but are consistently overlooked. He pointed out in particular that the question of causality should not be discussed in isolation, separated from the context in which decisions have to be made whether to act on the available evidence or not. He observed that,” it almost inevitably leads us to introduce differential standards before we convict. Thus on relatively slight evidence we might decide to restrict the use of a drug for early-morning sickness in pregnant women. If we are wrong in deducing causation from association no great harm will be done. The good lady and the pharmaceutical industry will doubtless survive. On fair evidence we might take action on what appears to be an occupational hazard, e.g. we might change from a probably carcinogenic oil to a non-carcinogenic oil in a limited environment and without too much injustice if we are wrong. But we should need very strong evidence before we made people burn a fuel in their homes that they do not like or stop smoking the cigarettes and eating the fats and sugar that they do like.” Indeed, Bradford Hill himself went as far as stating that “none of my nine viewpoints can bring indisputable evidence for or against the cause and effect hypothesis and none can be required as a sin qua non……what they can do is help us to make up our minds on the answer to the fundamental question – is there another way of explaining the set of facts before us”.

Thus, it will be important to make progress in this debate to have a rational and three-dimensional view of “causation” and to apply this view consistently. Finally, it is important to reach a consensus about how to “weigh” results of epidemiology studies against data collected in controlled exposure studies, and how to “weigh” epidemiology studies with different designs against one another. This will be discussed in more detail below.

Transparent, reproducible methods are needed for systematic reviews of EDCs. As noted in the introduction, two recent major reviews of the EDC literature were highlighted for the lack of systematic review of the literature. For example, Lamb et al. [12] concluded that the UNEP/WHO document [11] lacked a systematic approach to the literature to such a degree that it could not be considered a “state of the science” of EDCs. However, it would appear that Lamb and colleagues themselves do not always adhere to these standards. In 2007, two of the authors critiquing the UNEP/WHO document [11], Hentz and Lamb, published a document for the Weinberg Group entitled “2007 Update: State of the Science and Policy for Endocrine Disruption”, dated May 29, 2007 [Note: This document is no longer available on the internet, but on request, the authors are happy to provide the document to anyone interested]. This succinct (14 pages) report develops the theme on the basis of 21 references, and shows that it may well be possible to produce a state of the science document without a systematic approach to analyzing the literature. Discussions of this kind are largely futile and do nothing to resolve the impasse in the debate about endocrine disrupters. Lamb et al. [12] also concluded that techniques of systematic reviews are well established and that the recent US EPA review on non-monotonic dose-responses was both methodical and even-handed. However, a National Academy Committee concluded just the opposite; that the US EPA review was neither methodical nor even-handed in its approach, and recommended that the report be re-done [53].

It is perhaps human nature to find an analysis well performed when one agrees with the conclusion; likewise, it is easy to find fault with analytical procedures when one does not agree with the conclusion. Clearly, this is why it is important to develop an effective procedure for systematic reviews, and independent scientists at the National Toxicology Program and academic groups currently are in the process of developing the framework and detailed criteria for systematic reviews [54–56]. One essential element of systematic review is to evaluate the quality of the publication under consideration for inclusion [57]. However, evaluating the quality of the experimental design and methods requires reviewers with expertise in the specific area of research, and this issue is not often considered. Expert knowledge is central – and critical – to “weighing” the value of different studies with different designs. This is also the view presented in the UNEP/WHO report on EDCs, in the subchapter “Framework for evaluation of evidence for endocrine disruption in humans and wildlife” [11]. Thus, a significant amount of work remains to develop systematic review methods that are generally accepted.

A tool commonly used by risk assessors for assessing study quality, the Klimisch score [58], was developed by three industry toxicologists, writing that “Tests conducted and reported according to internationally accepted guidelines and in accordance with Good Laboratory Practices (GLP) should have the highest grade of reliability”, and thus are given the highest score. Use of the Klimisch scoring system, and the high evaluation of studies using GLP in general, are unfortunate examples of the conflation of high quality study reporting with high quality study design and execution.

As new tools are developed, it will be important to recognize that integration of data across multiple information streams (*in vitro*, laboratory animal, epidemiology, etc.) will be important [57], and that evaluating the quality and relevance of information across disciplines requires people expert in those disciplines. Once developed and shown to produce non biased assessments, systematic review methods should be used to assess the EDC literature. However, because current approaches to systematic reviews limit their use to a single chemical-disease dyad, a state of the science review may not be possible to complete using systematic review criteria because it would require hundreds (or more) of individual systematic reviews, followed by a meta-analysis of the systematic reviews, before any final conclusions could be reached. For example, although the 2002 IPCS document on EDCs discussed systematic reviews [16], it was only employed in Chapter 7 for the purpose of illustration and used endometriosis and TCDDs and/or PCBs; in addition, it lacked many of the elements being described currently by the NTP and NAS. In light of the absence of systematic review guidelines and the impossibility of using them for such a large undertaking, state of the science reviews are likely to always require the expertise of scientists working in the field and narrative reviews.

## Conclusions

There is intense scientific debate on the issue of EDCs that is not productive in its current form. We list here nine points that could provide a constructive path forward.

1. The definition of an EDC should focus on hormone action instead of hormone concentrations in blood. This would focus the debate on mechanisms of EDC effects rather than alterations in “homeostasis”.
2. An accepted definition of “adverse” is needed, along with more transparency in the ways in which particular endpoints are considered adverse (or “adaptive”). At this time, the IPCS/OECD definition of adverse is preferred as it includes not only direct/immediate responses to chemical exposure but also situations where the exposure results in a phenotype only in the presence of an additional environmental challenge or stressor.
3. The definition of the endocrine system should be that which emphasizes the role of hormones in development and the importance of timing of hormone action.
4. The potency of a substance is dependent on the endpoint. It is therefore important to agree on the endpoints to consider as metrics of “potency” and recognize that as new science becomes available our perception of the relative potency of chemicals may change.
5. Guideline studies rely on endpoints validated for reproducibility, not for their power to predict adverse effects in the human population. The current CLARITY-BPA study provides a mechanism by which new endpoints can be quickly validated for inclusion in guideline studies. In the meantime, the publically-funded, scientific literature must be included in any analysis of EDC effects.
6. There are currently three definitions of “low dose”; thus it is critical that the definition being used is noted in any related discussion. It is not acceptable to dismiss low dose effects simply because there is not one widely accepted definition.
7. The debate over whether EDC effects have a threshold, while scientifically interesting, cannot be proven or disproven with available technology. Thus, continuing this debate is not productive.
8. There is a need for agreement on the rules of evidence sufficient to conclude a causal relationship between environmental exposures and health outcomes. Although there are challenges to the use of the Hill approach for EDCs, agreeable adaptations could be made for use in this field.
9. It is important to develop transparent, consistent and unbiased criteria for the systematic review of EDCs. However, systematic review methods are currently used to address highly focused questions exploring chemical-disease dyads such as, does chemical X cause disease Y? It is therefore currently not possible to use systematic review criteria to answer broad questions that draw from all fields of endocrine disruption.

## Abbreviations

BP: Benzylparaben
BPA: Bisphenol A
EDCs: Endocrine Disrupting Chemicals
EE: 17α-ethinylestradiol
EPA: Environmental Protection Agency
FDA: Food and Drug Administration
GLP: Good Laboratory Practice
IPCS: International Programme on Chemical Safety
LD50: Lethal Dose 50%
LOAEL: Lowest Observed Adverse Effect Level
NAS: National Academy of Science
NIEHS: National Institute of Environmental Health Science
NOAEL: No Observed Adverse Effect Level
NTP: National Toxicology Program
OECD: Organization of Economic Cooperation and Development
PCB: Polychlorinated biphenyl
TCDD: Tetrachlorodibenzo-dioxin
UNEP: United Nations Environmental Programme
WHO: World Health Organization.
